# Sleeve gastrectomy causes weight‐loss independent improvements in hepatic steatosis

**DOI:** 10.1111/liv.15614

**Published:** 2023-05-19

**Authors:** Emma Rose McGlone, Matthieu Siebert, Marian Dore, David C. D. Hope, Iona Davies, Bryn Owen, Bernard Khoo, Rob Goldin, Dave Carling, Stephen Bloom, Maude Le Gall, Tricia M‐M. Tan

**Affiliations:** ^1^ Department of Surgery and Cancer Imperial College London London UK; ^2^ Department of Metabolism, Digestion and Reproduction Imperial College London London UK; ^3^ Centre de Recherche sur l'Inflammation, UMRS1149, Inserm, Université Paris Cité Paris France; ^4^ Genomics Facility MRC London Institute of Medical Sciences (LMS), Imperial College London London UK; ^5^ Division of Medicine University College London, Royal Free Hospital London UK; ^6^ Cellular Stress Group MRC LMS, Imperial College London London UK

**Keywords:** bariatric surgery, glucagon, non‐alcoholic fatty liver disease, weight loss

## Abstract

**Background and Aims:**

Sleeve gastrectomy (VSG) leads to improvement in hepatic steatosis, associated with weight loss. The aims of this study were to investigate whether VSG leads to weight‐loss independent improvements in liver steatosis in mice with diet‐induced obesity (DIO); and to metabolically and transcriptomically profile hepatic changes in mice undergoing VSG.

**Methods:**

Mice with DIO were treated with VSG, sham surgery with subsequent food restriction to weight‐match to the VSG group (Sham‐WM), or sham surgery with return to unrestricted diet (Sham‐Ad lib). Hepatic steatosis, glucose tolerance, insulin and glucagon resistance, and hepatic transcriptomics were investigated at the end of the study period and treatment groups were compared with mice undergoing sham surgery only (Sham‐Ad lib).

**Results:**

VSG led to much greater improvement in liver steatosis than Sham‐WM (liver triglyceride mg/mg 2.5 ± 0.1, 2.1 ± 0.2, 1.6 ± 0.1 for Sham‐AL, Sham‐WM and VSG respectively; *p* = 0.003). Homeostatic model assessment of insulin resistance was improved following VSG only (51.2 ± 8.8, 36.3 ± 5.3, 22.3 ± 6.1 for Sham‐AL, Sham‐WM and VSG respectively; *p* = 0.03). The glucagon‐alanine index, a measure of glucagon resistance, fell with VSG but was significantly increased in Sham‐WM (9.8 ± 1.7, 25.8 ± 4.6 and 5.2 ± 1.2 in Sham Ad‐lib, Sham‐WM and VSG respectively; *p* = 0.0003). Genes downstream of glucagon receptor signalling which govern fatty acid synthesis (*Acaca, Acacb, Me1, Acly, Fasn* and *Elovl6*) were downregulated following VSG but upregulated in Sham‐WM.

**Conclusions:**

Changes in glucagon sensitivity may contribute to weight‐loss independent improvements in hepatic steatosis following VSG.

**Abbreviations**
AGB: adjustable gastric band
DIO: diet induced obesity
HFD: high fat diet
HOMA‐IR: homeostatic model assessment of insulin resistance
NAFLD: non‐alcoholic fatty liver disease
NASH: non‐alcoholic steatohepatitis
RYGB: Roux‐en‐Y gastric bypass
Sham‐Ad lib: sham surgery with return to unrestricted diet
Sham‐WM: sham surgery with subsequent food restriction to weight‐match to the VSG group
T2D: type 2 diabetes mellitus
VSG: sleeve gastrectomy

**Key points**

- VSG leads to greater improvement in liver steatosis than equivalent weight loss by calorie restriction alone.
- VSG is associated with greater improvements in glucose tolerance and insulin resistance than equivalent weight loss by calorie restriction alone.
- VSG leads to an improvement in glucagon sensitivity as assessed by biochemical markers and hepatic transcriptomics.
- In contrast, glucagon sensitivity is reduced after equivalent weight loss by calorie restriction.

## 1 INTRODUCTION

Non‐alcoholic fatty liver disease (NAFLD) is closely linked to obesity and type 2 diabetes mellitus (T2D). It affects 25% of the global population and is a leading cause of liver‐related morbidity and mortality.^1^, ^2^ The best evidenced and most effective treatment for NAFLD is weight loss.^3^, ^4^ Bariatric operations are associated with excellent rates of resolution of NAFLD, durable for at least five years.^5^, ^6^, ^7^ Vertical sleeve gastrectomy (VSG) is as effective for NAFLD resolution as Roux‐en‐Y gastric bypass (RYGB)^8^, ^9^ and now accounts for more than half of primary bariatric procedures.^10^ As in humans, in rodents VSG leads to improvements in obesity‐related liver steatosis and steatohepatitis.^11^, ^12^, ^13^

It is well known that improvements in T2D and hypercholesterolaemia observed after bariatric surgery occur before or in the absence of significant weight loss.^14^, ^15^ For equivalent weight loss, RYGB and VSG lead to greater improvements in glucose homeostasis than calorie restriction or adjustable gastric band (AGB).^16^, ^17^, ^18^ The mechanisms underlying weight‐loss independent improvements in metabolic health are diverse and include altered gut and pancreatic hormone secretion, increased hepatic insulin sensitivity^19^, ^20^ and changes in adipose tissue distribution and function.^19^ To date, it is not known whether there is a weight loss‐independent component to NAFLD improvement seen after bariatric surgery. Studies of dietary weight loss have illustrated a relationship between extent of weight loss and likelihood of improvement in non‐alcoholic steatohepatitis (NASH), with 7% of total body mass considered necessary for appreciable benefit.^4^, ^21^ Meta‐analysis suggests that there is a dose‐relationship between weight loss and improvement in some domains of NAFLD for example inflammation and ballooning.^22^ In studies of both humans and rodents, however, histological improvement in NAFLD is greater following RYGB than AGB when weight‐loss differences are accounted for.^23^, ^24^

Hepatic resistance to glucagon signalling is observed in patients with increased liver fat.^25^ Since glucagon reduces liver fat by decreasing de novo lipogenesis and increasing fatty acid oxidation,^26^, ^27^ glucagon resistance may exacerbate hepatic steatosis.^28^, ^29^ Glucagon also stimulates hepatic ureagenesis, using non‐branched amino acids as substrates, thus reduced glucagon activity is indicated by increased fasting plasma alanine; in turn, increased plasma amino acids stimulate pancreatic alpha cells to release glucagon causing hyperglucagonaemia.^30^ A recent study indicates that glucagon resistance, as measured by the glucagon‐alanine index, the product of fasting plasma alanine and glucagon, may improve following bariatric surgery.^31^

The aims of this study were firstly to determine whether VSG leads to improvements in liver steatosis in mice with diet‐induced obesity that are greater than those due to weight loss alone; and secondly, to metabolically and transcriptomically profile hepatic changes in mice undergoing equivalent weight loss via VSG and calorie restriction.

## 2 METHODS

### 2.1 Animals

C57BL/6J male mice (Charles River, UK) were housed in cages at controlled temperature (22°C) with a 12 h light dark cycle with free access to water. All interventions were performed during the light cycle. Mice were weaned on standard chow pellets, which contain 11% kcal from fat, 27% from protein and 62% from carbohydrate (Special Diets Services RM3), then from 5 weeks of age were given free access to a high fat diet (Research Diets D12492) containing 60% kcal from fat (lard), 20% kcal from protein (casein) and 20% kcal from carbohydrate (maltodextrin and sucrose). Mice were housed in groups of 5 from birth until age 17 weeks, from which point they were individually housed to allow monitoring of food intake. At age 19 weeks mice that had failed to gain weight on the HFD or whose weight had failed to stabilise during the period of single‐housing (approximately 10%) were excluded, and remaining mice were allocated to study groups using stratified (by weight) random sampling. Surgical intervention and peri‐operative care are described below. Mice were returned to high fat diet until their weight stabilised at a similar weight to a reference group of mice maintained on standard chow throughout the study period.

### 2.2 Ethical approval statement

Experiments were performed in accordance with EU directives for animal experimentation and were approved by the Ethical Committee of Paris North and The French Minister of Higher Education, Research and Innovation (APAFIS #15884).

### 2.3 Surgical intervention and cull

Mice were allocated to the following groups, with seven or eight mice in each: either vertical sleeve gastrectomy (VSG), sham surgery with subsequent food restriction to weight‐match with the VSG group (Sham‐WM), or sham surgery (Sham‐Ad lib). Mice were introduced to gel diet (SAFE® gel diet energy, SAFE Complete Care Competence, Germany) 48 h before the operation and fasted overnight before the surgery. Surgery was performed under isoflurane anaesthesia, on a warming mat, using aseptic conditions (shaved and betadine swab). After midline laparotomy through the linea alba, the stomach was gently mobilised from surrounding tissues and a sleeve was fashioned by excluding 90% of stomach volume including the entire pro‐stomach and fundus with a curved clip, following which the compression line was cut along with fine tissue scissors. Stomach contents were gently removed with a cotton bud and the gastrotomy was closed using 8–0 Ethilon. Mass closure of the abdominal wall was with 5.0 prolene and skin with 7.0 prolene. Sham surgery consisted of laparotomy, mobilisation of stomach and gentle squeezing of stomach. All mice were given post‐operative xylocaine, co‐amoxiclav, buprenorphine and meloxicam. After recovery, mice were returned to their home cage in which gel diet was available immediately, with HFD returned the following day. One VSG mouse died on the second postoperative day due to a leak of the suture line.

Following weight stabilisation, mice were culled at day 16 via decapitation following a 5‐hour period of food deprivation to allow collection of tissues for further assay. Plasma was obtained from blood collected in tubes on ice that had previously been flushed with heparin 1000 IU/mL and contained 1 μL protease inhibitor (Sigma) and immediately spun at 4°C. Serum was obtained from blood, allowed to clot at room temperature for 10 min before spinning at 4°C. Samples were separated and aliquots of plasma and serum were stored at −80°C. Organs were harvested rapidly, weighed, and either snap frozen in liquid nitrogen or fixed in 2% paraformaldehyde for subsequent histopathological examination.

### 2.4 Hepatic histology and biochemistry

Lipids were extracted from tissue by homogenization in ethanol (volume of ethanol (mL) = liver weight (mg) × 0.03).^32^ Triglyceride content of samples was measured using a GPO‐PAP Triglyceride assay (Randox Laboratories Ltd, UK) and cholesterol using Amplex Red Cholesterol Assay Kit (ThermoFisher Scientific, UK). Liver histology was assessed using the Non‐alcoholic fatty liver disease Activity Score (NAS)^33^ by a pathologist with a special interest in liver disease and blinded to treatment assignment, using haematoxylin and eosin and Sirius Red‐stained paraffin sections.

### 2.5 Clinical chemistry

Plasma from mice after 5‐hour food restriction was assayed using ultra‐sensitive mouse insulin, leptin and adiponectin enzyme‐linked immunosorbent assay kits (Crystal Chem, Netherlands). Glucose was measured using the glucose oxidase‐PAP method (Randox) and glucagon with the Meso Scale Discovery (MSD, USA) system. L‐amino acids, alanine and branched chain amino acids were measured with colorimetric assays from Sigma‐Aldrich. All assays had inter‐ and intra‐assay CVs of ≤10%.

Homeostatic model assessment of insulin resistance (HOMA‐IR) was calculated using the formula (fasting glucose mmol/L × fasting insulin mU/L)/22.5.^34^

Serum transaminases were assayed at the Core Biochemical Assay Laboratory, Cambridge, UK and serum triglyceride using a GPO‐PAP Triglyceride assay (Randox).

### 2.6 Glucose tolerance testing

Glucose tolerance testing was performed following weight stabilisation at day 14 in mice following 5‐hour food restriction. Blood was sampled from the tail vein before and at time intervals following intraperitoneal injection of 20% glucose (2 mg/g lean body weight, estimated to be 31 g based on the mean weight of a cohort of mice from the same batch fed standard chow throughout the study period). Glucose was measured using a calibrated glucometer (GlucoRx Nexus, UK). iAUC was calculated from baseline glucose reading to account for differences in fasting glucose.

### 2.7 RNA isolation, targeted‐qPCR, RNA sequencing and data analysis

Total RNA was extracted from snap‐frozen liver tissue using TRIzol reagent (ThermoFisher Scientific, UK) prior to mechanical homogenisation. Chloroform was then added to samples before centrifugation at 16 000 *g* for 15 min. Supernatant was added to 70% iso‐propanol in a 1:1 mixture and left at room temperature for 10 min to precipitate RNA. Samples were then centrifuged at 16 000 *g* for a further 15 min and the resulting RNA pellet was washed with 70% ethanol before re‐suspending in RNase‐free water.

For RT‐qPCR, cDNA synthesis was carried out using a commercially available kit (iScript cDNA synthesis kit, BioRad). Targeted qPCR was then carried out using the SYBR Green method and KiCqStart SYBR Green primers m_Fasn_1, m_scd_1, m_elovl6_1, m_acly_1 (Merck). For RNA sequencing, RNA quality was assessed using the Agilent 2100 Bioanalyser RNA 6000 Nano assay: only samples with RNA‐integrity number of >7.5 were used for further analysis. Preparation of RNA‐seq libraries, library QC, and subsequent NGS sequencing was carried out by Laurence Game and Ivan Andrew of the LMS Genomics Facility (Hammersmith Hospital, London). Libraries were prepared using the NEBNext Ultra II Directional RNA Library Prep Kit with NEBNext Poly(A) mRNA Magnetic Isolation Module. Library quality was evaluated on a Bioanalyser HS DNA chip and the concentrations were measured using the Qubit™ dsDNA HS Assay Kit. Libraries were pooled in equimolar quantities and sequenced on a Hiseq2500 to generate a minimum of 20 million Paired End 100 bp reads per sample. Reads were aligned to UCSC mouse genome (mm10) using tophat2 version 2.1.1.^35^ Mapped reads that fell on genes were counted using featureCounts from the Rsubread package 1.28.1, using R version 3.4.3.^36^ Generated count data were then used to normalise and identify differentially expressed genes as compared to the control for the experiment (Sham‐Ad lib) using DESeq2,^37^ which were defined with Benjamini‐Hochberg adjusted *p* < 0.05.

Gene Set Enrichment Analysis was performed using GSEA version 4.1.0^38^ on pre‐ranked lists generated by DESeq2 version 1.34.0. Unwanted variation due to batch was removed from mice in surgical cohorts using RUVSeq version 1.28.0^39^ with *k* = 2. The Qiagen Ingenuity Pathway Analysis software was used to explore the differential expression of genes in the Sham‐WM and VSG groups, compared to the Sham‐Ad lib group. As noted in the text, log2 of fold expression changes of >1 or <−1 for VSG versus Sham Ad lib were considered biologically significant, filtered by *p* or adjusted *p*‐values (*q*‐values or false discovery rate) of <0.05.

### 2.8 Statistical analyses

Unless otherwise stated, data are presented as mean and standard error of mean. GraphPad Prism 9.3.1 (GraphPad Software, USA) was used for graphing and statistical analysis. Datasets were compared using one‐way ANOVA, followed by Tukey's multiple comparison post‐hoc tests. For comparison of non‐normally distributed data, a Kruskal–Wallis test with Dunn's multiple comparison post‐hoc test was used. Comparisons were made with reference to the control group (Sham‐Ad lib) and the calorie‐restricted group (Sham‐WM) for each intervention.

## 3 RESULTS

### 3.1 VSG is associated with weight loss‐independent improvements in hepatic steatosis

**FIGURE 1:**
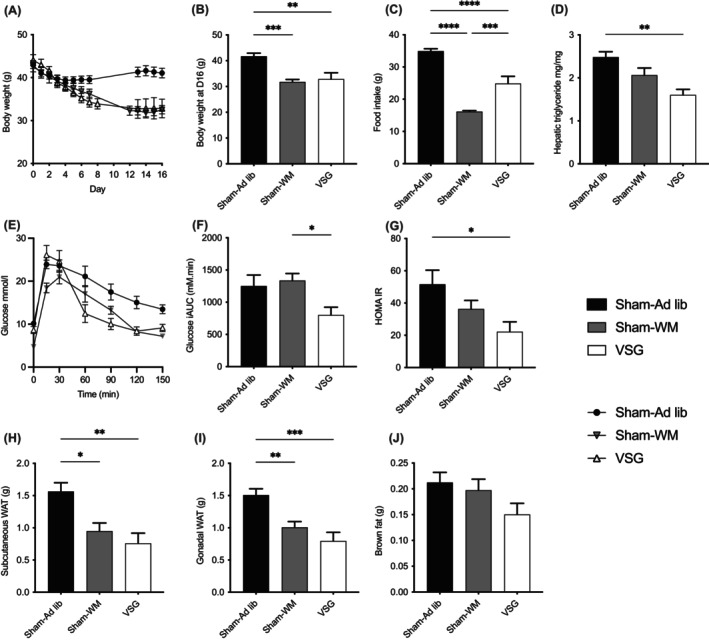
Sleeve gastrectomy leads to partially weight loss‐independent improvements in liver steatosis and glucose tolerance. Following 14 weeks on a high fat diet (HFD), obese mice were subjected to sham surgery with a return to HFD (Sham‐Ad lib; black), vertical sleeve gastrectomy (VSG; white) or sham surgery and food restriction to match the weight of VSG‐treated mice (Sham‐WM; grey). (A) Weight trajectory over time. (B) Final weight at day 16. (C) Cumulative food intake over study period. (D) Hepatic triglyceride at end of study. (E) Intra‐peritoneal glucose tolerance test (IPGTT); (F) iAUC_0‐150_ during IPGTT. (G) Homeostatic model assessment of insulin resistance (HOMA‐IR) calculated from 5‐hour fasted glucose and insulin levels at end of study period. (H–J) Tissue weights at end of study period. WAT, white adipose tissue. *n* = 6–8 in each group, mean and SEM; one‐way ANOVA and Tukey's post‐hoc test: *p < 0.05, **p < 0.01, ***p < 0.001 when compared with Sham‐Ad lib.

To determine if the effects of sleeve gastrectomy (VSG) on hepatic steatosis are weight‐loss dependent, we subjected obese mice to either VSG, sham surgery with subsequent food restriction to weight‐match to the VSG group (Sham‐WM), or sham surgery with return to unrestricted diet (Sham‐Ad lib). Over the treatment period of 16 days, mice in the VSG cohort and Sham‐WM groups lost around 25% of their body weight, stabilising for the final few days at a weight equivalent to a reference group of mice from the same cohort maintained on standard chow throughout the study period (Table 1; Figure 1A,B). Although weight loss was equivalent between the two treatment groups, mice treated with VSG had a significantly greater cumulative food intake than Sham‐WM mice over this period (Figure 1C).

**TABLE 1:** Body weight and biomarkers of NAFLD for mice at the end of the study period.

	Sham‐Ad lib	Sham‐WM	VSG	Standard chow^a^
Weight at end of study period (g)	41.7 ± 1.2	31.8 ± 0.9***	32.9 ± 2.5**	31.7 ± 0.5
Non‐alcoholic fatty liver disease Activity Score; median (interquartile range)	3 (2–3)	1 (0–1.75)^$^	1 (0–2)^$^	0 (0–0)
Hepatic triglyceride (mg/mg)	2.5 ± 0.1	2.1 ± 0.2	1.6 ± 0.1**	1.7 ± 0.2
Hepatic cholesterol (μg/mg liver)	2.0 ± 0.1	2.5 ± 0.2	2.5 ± 0.3	3.0 ± 0.3
Alkaline phosphatase (U/L)	61 ± 3.9	65 ± 3.9	62 ± 2.9	74.4 ± 1.7
Alanine aminotransferase (U/L)	68 ± 9.9	81.3 ± 13.4	69 ± 6.5	58.1 ± 3.7

*Note*: Mice culled at the end of the treatment period after 5‐hour food deprivation. Medians compared with Kruskal‐Wallis test with Dunn's post‐hoc test.

^a^ Standard chow‐fed mice (*n* = 10) of the same age are included for reference but have not been included in statistical analysis. Mean and SEM unless otherwise stated; comparisons made with one‐way ANOVA and Tukey's multiple comparison test.

** *p* < 0.01

*** *p* < 0.001 for comparison with Sham‐Ad lib;

^$^ *p* < 0.05 for comparison with Sham‐Ad lib.

At the end of the study period liver triglyceride content decreased in Sham‐WM mice but was statistically significantly reduced in VSG mice only (Figure 1D, Table 1). Liver triglyceride in mice treated with VSG was at similar levels as that seen in mice from the same cohort maintained on standard chow throughout. Non‐alcoholic fatty liver disease Activity Score (NAS) was reduced in mice treated with both VSG and Sham‐WM (Table 1; Supporting Information Figure S1). Sham‐Ad lib mice had mild pericellular fibrosis (Stage 1a) as can be anticipated after treatment with a HFD^33^; mice from the other treatment groups had no significant fibrosis, consistent with overall improvement in their hepatic steatosis and NAS (Supporting Information Figure S1). Hepatic enzymes (alkaline phosphatase and alanine aminotransferase) were at similar levels to those measured in mice maintained on standard chow (Table 1).

### 3.2 VSG is associated with weight loss‐independent improvements in insulin resistance

The reductions in body weight seen in both VSG and Sham‐WM mice were associated with changes in glucose tolerance (Figure 1E), and a decreased incremental glucose area under the curve following intra‐peritoneal glucose injection in mice treated with VSG (Figure 1F). Fasting serum insulin numerically decreased in mice treated with VSG (1.4 ± 0.3 vs. 2.2 ± 0.3 pmoL/L; *p* = 0.08; Table 2) and homeostatic model assessment of insulin resistance (HOMA‐IR) was significantly reduced in VSG mice only (Figure 1G; Table 2).

**TABLE 2:** Fasting blood biochemistry for mice at the end of the study period.

	Sham‐Ad lib	Sham‐WM	VSG
Triglyceride (mg/dL)	61.3 ± 3.7	91.6 ± 11.5	73.4 ± 15.2
Glucose (mmol/L)	18.2 ± 1.7	11.9 ± 0.6*	12.3 ± 1.5*
Insulin (pmol/L)	2.2 ± 0.3	2.1 ± 0.3	1.4 ± 0.3
HOMA‐IR	51.2 ± 8.8	36.3 ± 5.3	22.3 ± 6.1*
Total amino acids (mmol/L)	5.1 ± 0.4	4.8 ± 0.4	3.9 ± 0.4
Branched‐chain amino acids (mmol/L)	1.2 ± 0.2	1.8 ± 0.3	0.6 ± 0.1^^

*Note*: Mice culled at the end of the treatment period after 5‐hour food deprivation. Mean and SEM; one‐way ANOVA and Tukey's multiple comparison test.

* *p* < 0.05

^^^^ *p* < 0.01 for comparison with Sham‐WM.

**FIGURE 2:**
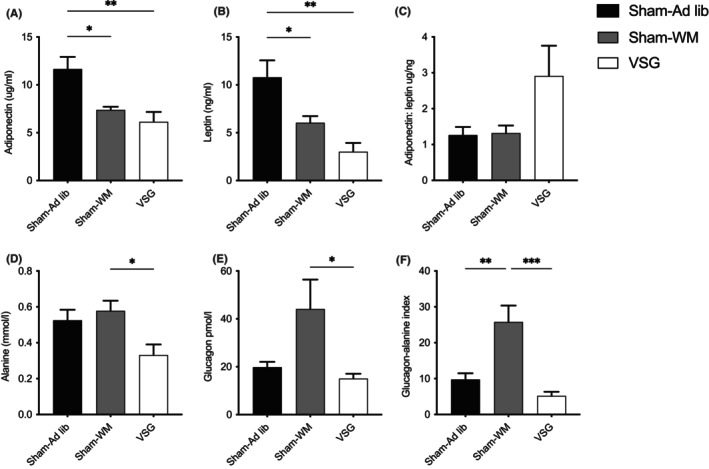
Sleeve gastrectomy is associated with changes in circulating metabolic hormones. Following 14 weeks on a high fat diet, obese mice were subjected to sham surgery with a return to HFD (Sham‐Ad lib; black), vertical sleeve gastrectomy (VSG; white) or sham surgery and food restriction to match the weight of VSG‐treated mice (Sham‐WM; grey). (A,B) Serum leptin and adiponectin. (C) Adiponectin: leptin ratio. (D,E) Plasma alanine and glucagon. (F) Glucagon‐alanine index. 5‐hour fasting levels and the end of the study period (16 days). *n* = 6–8 in each group, mean and SEM. *p < 0.05, **p < 0.01, ***p < 0.01 (one‐way ANOVA and Tukey's post‐hoc test).

Subcutaneous and gonadal white adipose tissue stores were significantly reduced in both VSG and Sham‐WM mice, with brown fat mass not affected (Figure 1H–J). Circulating adiponectin was reduced in both treatment groups, and leptin fell to a greater extent in VSG than Sham‐WM mice (Figure 2A,B). The adiponectin‐leptin ratio, a marker of insulin sensitivity and overall adipose tissue health, was increased in mice treated with VSG (*p* = 0.06; Figure 2C).^40^

Numerically lower fasting amino acids following VSG reflected significant reductions in branched‐chain amino acids (Table 2), as well as alanine (Figure 2D). Mice treated with VSG exhibited a trend to reduction in markers of glucagon resistance, namely, fasting alanine and glucagon‐alanine index, when compared to Sham‐Ad lib (0.33 ± 0.06 vs. 0.52 ± 0.06; *p* = 0.08, and 5.2 ± 1.6 vs. 10.1 ± 2.0; *p* = 0.09 respectively: Figure 2D,F). Conversely, mice treated with Sham‐WM had higher fasting alanine and glucagon than mice treated with VSG, resulting in a higher glucagon‐alanine index.

### 3.3 Sleeve gastrectomy drives weight loss‐independent hepatic transcriptomic changes

**FIGURE 3:**
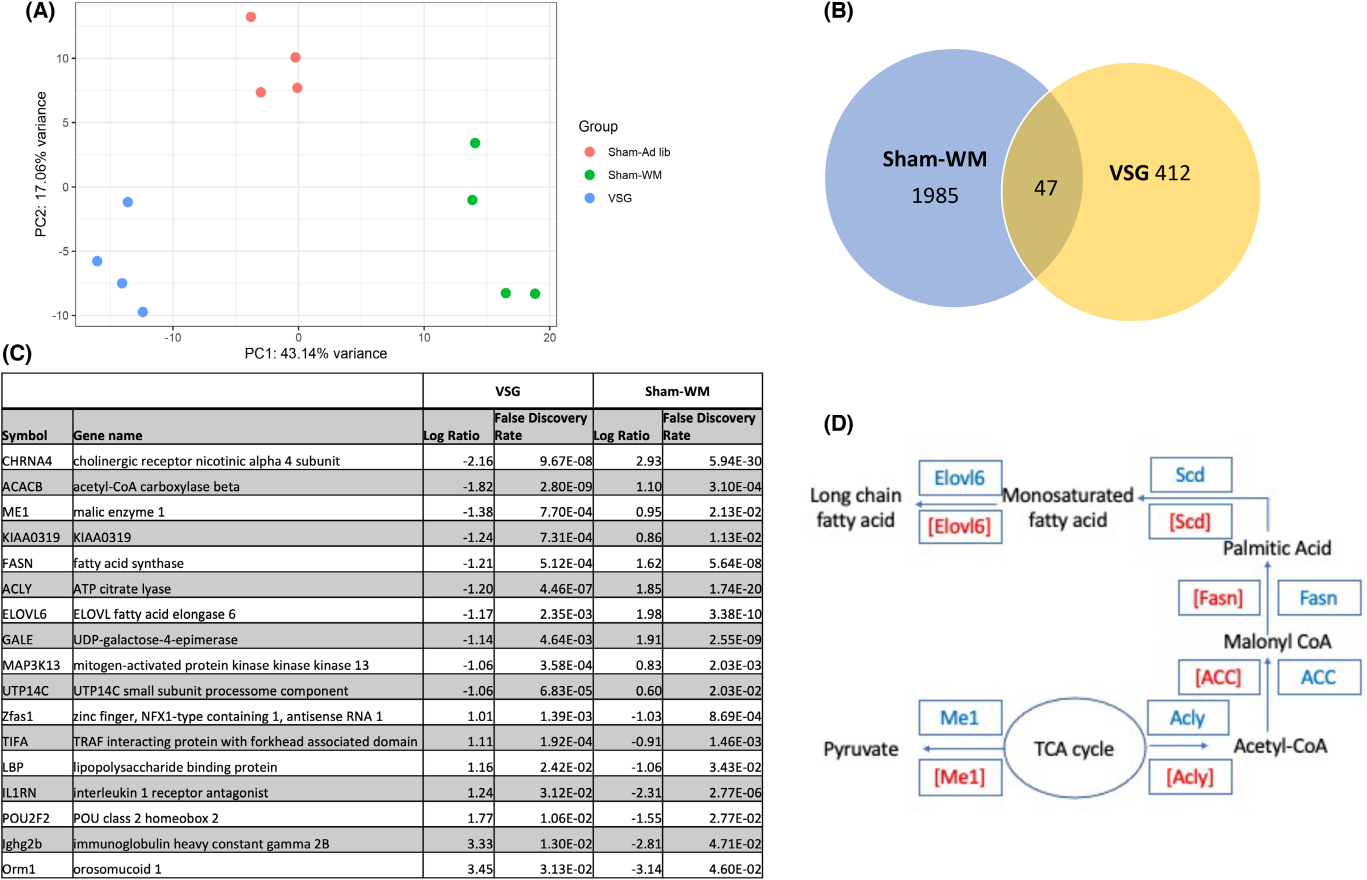
Sleeve gastrectomy drives improvement in hepatic steatosis by downregulating hepatic metabolic pathways which are distinct to those altered by weight loss alone. (A) 2D PCA of hepatic transcriptomic data of surgically treated mice. (B) Differential gene expression in livers of mice treated with Sham‐WM and VSG. *q* value <0.05 for each treatment versus Sham‐Ad lib. (C) Discordant genes in VSG and Sham‐WM versus Sham‐Ad lib, filtered for log ratio >1 or <1 for VSG and *q* < 0.05 in both. (D) Pathway schema indicating differences in triglyceride metabolism following VSG when compared to Sham‐WM, blue indicates downregulation and red is upregulation; normal script is VSG while [] indicates Sham‐WM treatment.

To investigate whether hepatic transcriptomic pathways were similarly affected by VSG and equivalent weight loss by calorie restriction, we performed RNA‐sequencing of liver tissue from mice at the end of the study period. Unsupervised principal component analysis demonstrated clear separation between gene expression profiles of mice treated with VSG compared to Sham‐WM (Figure 3A). When comparing the treatment groups to Sham‐Ad lib, we found many differentially expressed genes (*p* < 0.05 in both groups) although there was little overlap in changes following VSG and those following Sham‐WM (Figure 3B). Of the 459 genes affected by VSG, 47 (10%) were similarly up‐ or down‐regulated in mice treated by Sham‐WM.

Seventeen genes were statistically and biologically significantly changed after both treatments when compared to Sham‐Ad lib, but in different directions (*q* < 0.05 for both treatments; log2 ratio >1 or <−1 for VSG versus Sham‐Ad lib) (Figure 3C). Downregulated genes after VSG were primarily those related to lipid metabolism, with levels of acetyl‐CoA carboxylase (*ACC*) Beta (*Acacb)*, Malic enzyme 1 *(Me1)*, ATP citrate lyase *(Acly)*, Fatty acid synthase *(Fasn)*, and Elongation of very long chain fatty acids protein 6 (*Elovl6)* downregulated in the VSG group and upregulated in the Sham‐WM group. The alpha ACC isoform (*Acaca*) was also downregulated after VSG and upregulated following Sham‐WM (log ratio −0.9, *q* = 0.003 and log ratio 0.9 *q* = 0.002 respectively). Inhibition of lipogenesis is a key role of glucagon signalling in the liver and these genes for fatty acid synthesis are all targets of glucagon signalling^26^, ^28^, ^41^ (Figure 3D). A further key related gene, stearoyl‐CoA desaturase (*Scd*) was also significantly downregulated following VSG and upregulated in Sham‐WM but did not quite reach statistical significance in the latter group (VSG: log ratio −1.1, *q* = 0.003; Sham‐WM: log ratio 0.9, *q* = 0.056). To confirm these results we performed RT‐qPCR on newly extracted RNA for selected key fatty acid synthesis genes and found the same pattern, whereby levels diverged in the two treatment groups. All genes tested (*Fasn*, *Scd*, *Elov6* and *Acly)*, were upregulated in Sham‐WM mice with levels that were significantly greater than those seen in VSG mice (Supporting Information Figure S2).

UDP‐galactose‐4‐epimerase (*Gale*) was also downregulated in VSG mice but upregulated in Sham‐WM mice: this enzyme interconverts UDP‐galactose with UDP‐glucose and is a critical enzyme in hepatic gluconeogenesis^42^; in mouse‐models genetic reduction of the levels of this enzyme improves whole‐body glucose tolerance.^43^ Mitogen‐activated protein 3 kinase 13 (*Map3k13*) was also differentially regulated by the two weight‐loss treatments: this enzyme is implicated in JNK signalling, which is a mediator of hepatic Gi (negative) glucagon signalling.^44^ Enzymes upregulated in VSG and downregulated in Sham‐WM mice were mostly related to innate immunity.

## 4 DISCUSSION

It has previously been observed that in rodents with diet‐induced obesity, VSG leads to better steatosis outcomes than pair‐feeding, although VSG‐treated animals also lose more weight.^11^, ^45^ Here using a weight‐matched cohort design, we demonstrate that sleeve gastrectomy in mice with hepatic steatosis is associated with greater reduction in hepatic triglyceride content than that attributable to weight loss alone.

Another recent study also revealed superior improvements in steatosis in mice treated with VSG compared to a weight‐matched group: in this study mice were culled at 40 days post‐surgery, having regained more than 50% of the weight originally lost.^46^ Our study findings are also congruent with a small number of rodent studies in which a group undergoing VSG have ended at a similar weight to a control group, albeit not by design.^13^, ^23^ These studies terminated when animals had regained the weight lost following VSG, which is a potential problem because liver fat content is highly sensitive to overall weight gain or loss.^47^, ^48^ In our study we chose to cull during the stable nadir period of weight loss, to better model the clinical situation. In a non‐randomised prospective cohort study comparing RYGB with AGB in patients with NAFLD, hepatic histological improvement was more significant following RYGB.^24^ Multivariate analysis revealed this superiority to be primarily accounted for by greater weight loss, but there was an independent contribution from RYGB itself: this suggests that RYGB, but not AGB, has weight loss‐independent metabolic effects that are beneficial for NAFLD. A structured exercise programme has recently been shown to have greater benefits for liver steatosis, despite minimal weight loss, than a conventional weight loss programme in Japanese men.^49^ Our findings therefore add to the evidence that weight loss is not the only treatment for NAFLD and alternative mechanisms deserve urgent attention, especially given the fact that there are no approved pharmaceutical treatments specifically for NASH.^50^, ^51^

We found that insulin resistance improved to a greater degree in mice treated with VSG than following equivalent weight loss by dietary restriction alone. Hepatic insulin resistance is highly associated with NAFLD.^52^ Notably, it is characterised by selective reduction in insulin's hypoglycaemic effects with minimal change to its effects on lipogenesis.^53^ In our study, superior improvements in liver fat in VSG mice may have led to greater improvements in hepatic insulin sensitivity when compared to Sham‐WM. Alternatively, or additionally, better peripheral insulin sensitivity in mice treated with VSG may have decreased peripheral lipolysis and improved peripheral glucose uptake, thereby reducing the substrates available for hepatic fat deposition.^54^

Whilst insulin resistance is a well‐known pathogenic mechanism, the role of glucagon resistance has been underappreciated. Glucagon is a powerful regulator of hepatic metabolism: it increases glucose production, increases fat oxidation, reduces *de novo* lipogenesis and stimulates ureagenesis.^55^ Increases in plasma amino acids such as alanine signal to the alpha cell to increase glucagon secretion which then triggers hepatic uptake and oxidation of amino acids, increased ureagenesis to dispose of the nitrogen from amino acid oxidation, and consequently a drop in plasma amino acids, forming a physiological feedback cycle, the ‘liver‐alpha cell axis’.^30^ Analogous to insulin resistance, glucagon resistance is indicated by simultaneous hyperglucagonaemia and hyperaminoacidaemia, signifying pathophysiological disturbance of the liver‐alpha cell axis, and this can be quantified by a glucagon‐alanine index (product of fasting glucagon and alanine concentrations). Glucagon resistance is correlated with increased liver fat.^25^, ^56^ To date there exists very little data analysing the effect of weight loss on glucagon resistance. Our study is consistent with a recent clinical study indicating that glucagon sensitivity may improve following bariatric surgery.^31^ In our experimental setting, improved glucagon sensitivity was observed in mice treated with VSG but not in those experiencing an equivalent degree of weight loss by calorie restriction alone, which merits further investigation as a potential mechanism for the superior effects of VSG over calorie restriction on steatosis. One possible explanation is that VSG is better modelled by calorie dilution than calorie restriction. This hypothesis would be supported by the substantially greater food intake in VSG mice to achieve similar weight loss to those treated with Sham‐WM: VSG is likely to cause malabsorption, albeit to a lesser extent than RYGB, due to increased intestinal transit.^57^ Calorie restriction has been shown to have distinctive effects on mouse physiology when compared to calorie dilution.^58^ It results in hungrier mice, as assessed by behavioural assays, and a hypothalamic gene‐expression profile characteristic of starvation. A possible explanation for the findings of the present study, then, is that weight matching using calorie restriction causes an increase in fasting glucagon levels commensurate with a starvation state when compared to VSG.^59^

Our transcriptomic findings mirror our biochemical findings, as we observed that pathways relating to fatty acid production were reversed depending on treatment modality (VSG or Sham‐WM). These involved key genes downstream of glucagon‐stimulated lipid metabolism. Our findings are in line with those of other studies of VSG in mice: for example, *Fasn*, a rate‐limiting enzyme catalysing the de novo biosynthesis of long chain fatty acids, is downregulated following VSG but not weight loss by calorie restriction.^13^, ^60^, ^61^ Similarly *Acac* enzymes, which convert acetyl‐CoA to malonyl‐CoA, have also been reported to be uniquely downregulated following VSG.^13^, ^60^ Apart from *Gale*, a regulator of hepatic gluconeogenesis, none of the genes identified as discordantly expressed following the two treatments related to glucagon's other main actions of gluconeogenesis and ureagenesis. This may be because glucagon resistance affects metabolic pathways differentially^25^, ^29^; or because other hormonal and metabolic pathways compensate to a greater extent for some actions of glucagon than others. Changes in expression of hepatic genes related to immunity has previously been observed following VSG in mice.^60^

In conclusion, this study demonstrates that VSG leads to much greater improvement in liver steatosis than equivalent weight loss by calorie restriction; and that this is associated with superior improvements in glucose tolerance and adipokine hormone profile. Additionally, we present biochemical and transcriptomic evidence that changes in glucagon signalling are directionally opposite following weight loss via VSG when compared to calorie restriction alone. Our findings suggest that changes in glucagon sensitivity may contribute to weight‐loss independent improvements in NAFLD following VSG. Further studies are warranted to directly investigate changes in hepatic glucagon signalling following different weight loss treatments, for example using pancreatic clamps,^25^ in both mice and humans.

## AUTHOR CONTRIBUTIONS

Conceptualisation: Emma Rose McGlone, Dave Carling, Stephen Bloom, Maude Le Gall, Tricia Tan. Methodology: Emma Rose McGlone, Matthieu Siebert, Maude Le Gall; Investigation: Emma Rose McGlone, Matthieu Siebert, Marian Dore, David C. D. Hope, Bryn Owen, Rob Goldin, Bernard Khoo, Maude Le Gall, Tricia Tan. Writing – original draft: Emma Rose McGlone. Writing – review and editing: all authors.

## FINANCIAL DISCLOSURE

ERM was supported by a UK Medical Research Council (MRC) Clinical Research Training Fellowship while working on this project. MS was supported by Fondation pour la Recherche Médicale while working on this project. DCDH is supported by a UK Medical Research Council (MRC) Clinical Research Training Fellowship. MLG received support from the Société Francophone du Diabète. DC is funded by the MRC (MC‐A654‐5QB10). BK is funded by the J.P. Moulton Charitable Foundation. TT is funded by the NIHR, the NIHR Imperial BRC and the J.P. Moulton Charitable Foundation. SRB is funded by the NIHR Imperial BRC. The Department of Metabolism, Digestion and Reproduction at Imperial College London, UK, is funded by grants from the MRC, the BBSRC and is supported by the NIHR Imperial BRC. The views expressed are those of the authors and not necessarily those of the abovementioned funders, the UK National Health Service (NHS), the NIHR, or the UK Department of Health.

## CONFLICT OF INTEREST STATEMENT

The authors do not have any disclosures to report.

## Supporting information


Supplementary Figure 1.

## Data Availability

The data that support the findings of this study are openly available in Gene Expression Omnibus at https://www.ncbi.nlm.nih.gov/geo/, reference number GSE225843.
