# Supplementary material for: Sleeve gastrectomy causes weight‐loss independent improvements in hepatic steatosis
Source: Liver Int. 2023 May 19;43(9):1890–900. doi: 10.1111/liv.15614 (PMC10947097; doi:10.1111/liv.15614)
Supplement: Supplementary file 1 — Supplementary Figure 1. [file LIV-43-1890-s001.docx]

**Supplementary Figure 1:** Haematoxylin and Eosin (H&E), and Sirius Red stained liver sections from a mouse in the Sham-Ad lib group (A and B, respectively), from the Sham-WM group (C and D, respectively) and from the VSG group (E and F, respectively) (original magnifications x150). Sham-Ad lib mouse (A) demonstrates large droplet fatty change (starred) and ballooning (arrowed); sections from the other groups show none of these changes. Similarly (B) demonstrates mild pericellular fibrosis (NASH CRN 1a) while the other 2 groups show no fibrosis.


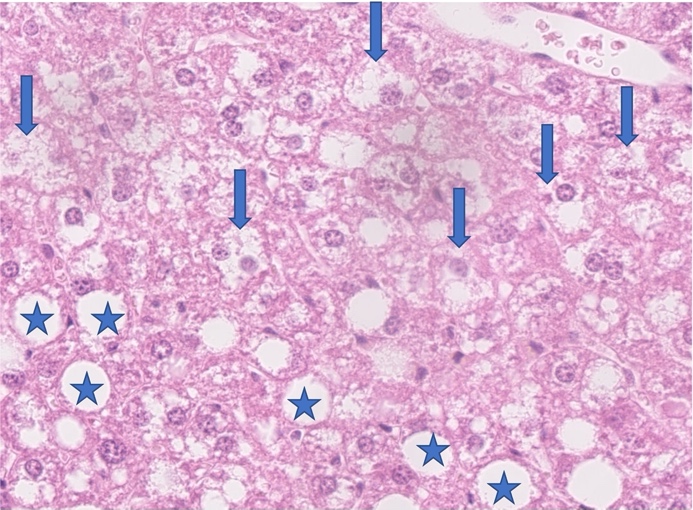

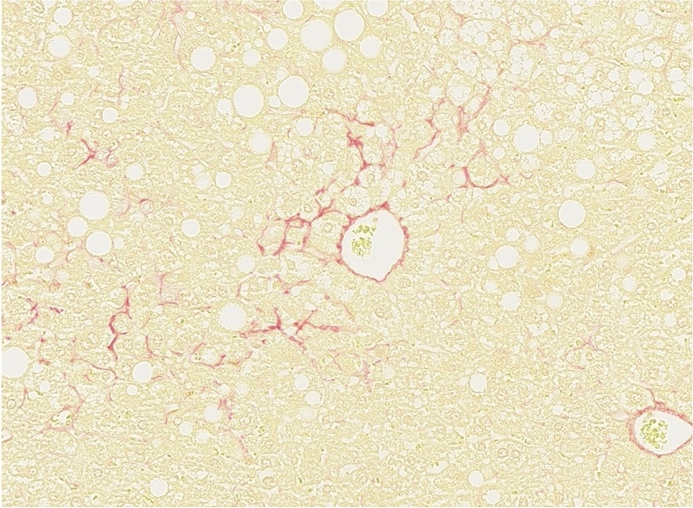

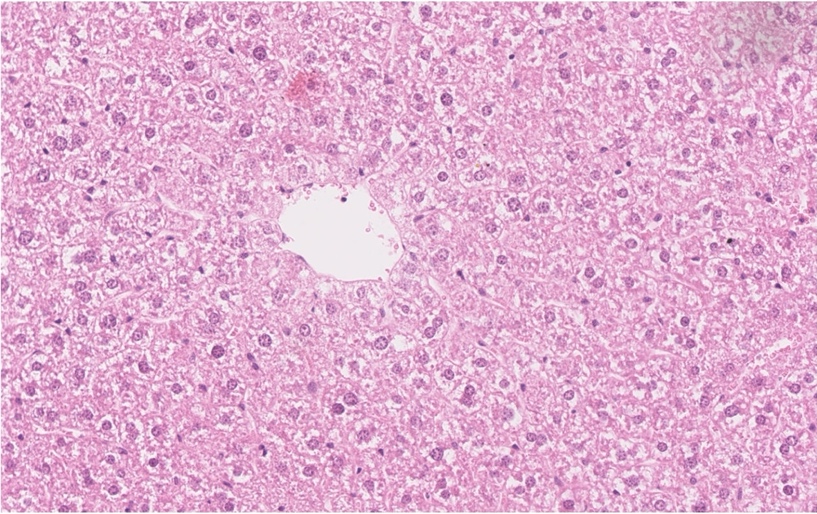

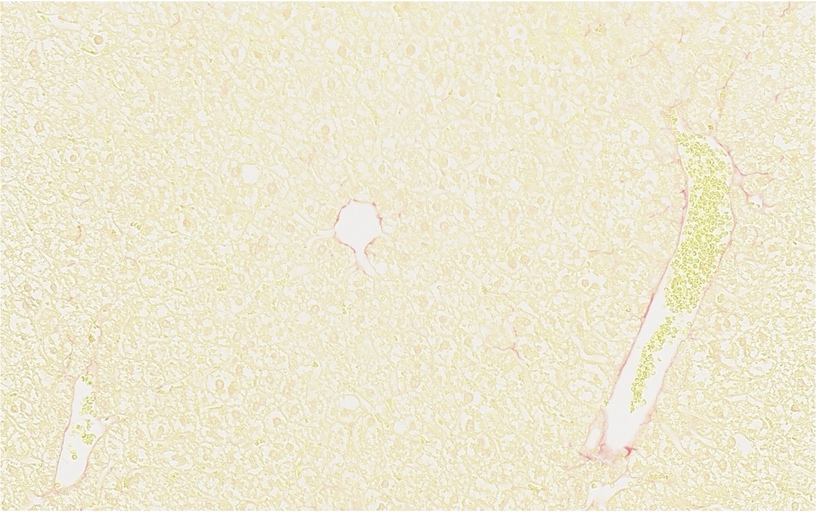

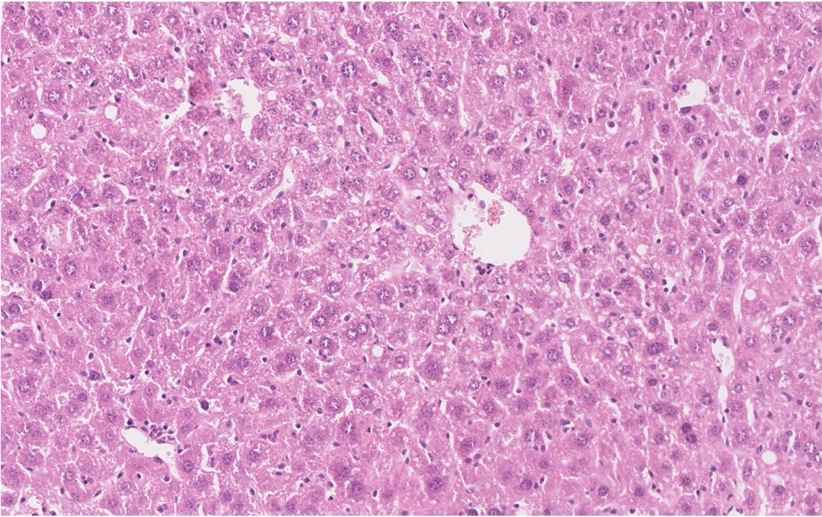

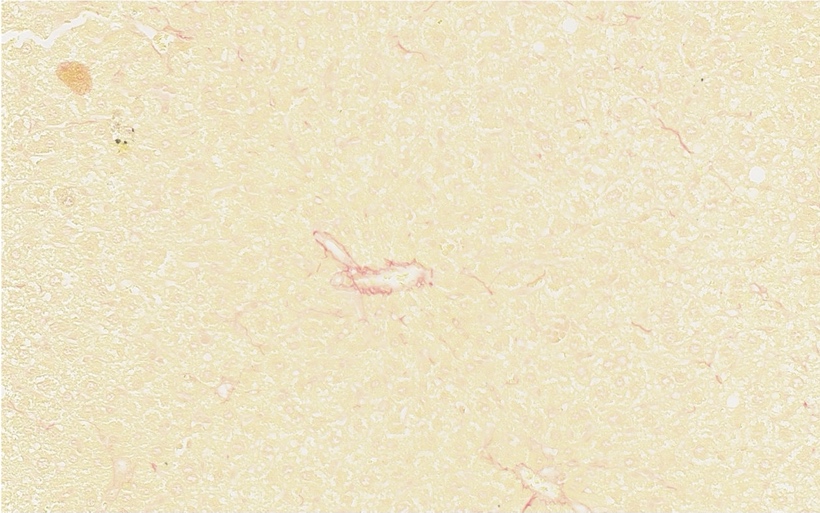


**F**

**E**

**D**

**C**

**B**

**A**

**Supplementary Figure 2:** Fatty acid synthesis genes expressed as fold change to level in Sham-Ad lib mice: A: Fatty acid synthase *(Fasn);* B: Stearoyl-CoA desaturase (*Scd*); C: Elongation of very long chain fatty acids protein 6 (*Elovl6)*; D: ATP citrate lyase *(Acly)*: data is mean **±** SEM; n = 5-7 in each group. Statistical significance was analysed using one-way ANOVA with Tukey’s multiple comparison test, ** p<0.01, *** p<0.001.
